# Correlations between the Quality of Life Domains and Clinical Variables in Sarcopenic Osteoporotic Postmenopausal Women

**DOI:** 10.3390/jcm9020441

**Published:** 2020-02-06

**Authors:** Mariana Cevei, Roxana Ramona Onofrei, Felicia Cioara, Dorina Stoicanescu

**Affiliations:** 1 Psychoneuro Sciences and Rehabilitation Department, Faculty of Medicine & Pharmacy, University of Oradea, 410087 Oradea, Romania; cevei_mariana@yahoo.com (M.C.); felicia_cioara@yahoo.com (F.C.); 2 Department of Rehabilitation, Physical Medicine and Rheumatology, “Victor Babeş” University of Medicine and Pharmacy Timișoara, 300041 Timișoara, Romania; 3 Microscopic Morphology Department, “Victor Babeş” University of Medicine and Pharmacy Timișoara, 300041 Timișoara, Romania; dstoicanescu@yahoo.com

**Keywords:** sarcopenia, quality of life, osteoporosis, postmenopausal women

## Abstract

(1) Background: both sarcopenia and osteoporosis are major health problems in postmenopausal women. The aim of the study was to evaluate the quality of life (QoL) and the associated factors for sarcopenia in osteoporotic postmenopausal women, diagnosed according to EWGSOP2 criteria. (2) Methods: the study sample comprised 122 osteoporotic postmenopausal women with low hand grip strength and was divided into two groups: group 1 (probable sarcopenia) and group 2 (sarcopenia). QoL was assessed using the validated Romanian version of SarQol questionnaire. (3) Results: the D1, D4, D5, D7 and total SarQoL scores were significantly lower in women from group 2 compared to group 1. In group 2, women older than 70 years had significant lower values for D1, D3, D4, D6 and total SarQoL scores. Age, history of falls and the presence of confirmed and severe sarcopenia were predictors for overall QoL. (4) Conclusions: the frequency of sarcopenia was relatively high in our sample, with body mass index and history of falls as predictors for sarcopenia. Older osteoporotic postmenopausal women, with previous falls and an established sarcopenia diagnosis (low muscle strength and low muscle mass), were more likely to have a decreased quality of life.

## 1. Introduction

Sarcopenia is characterized by decreased muscle strength, loss of muscle mass and poor physical performance [1]. The condition is associated with aging. Aging is a complex process, involving many variables that interact with each other and include, besides genetic factors, lifestyle and chronic diseases. Even if sarcopenia is more common among older individuals, it can also occur earlier in life. It typically begins in the fourth decade of life, but the decline is accelerated after the sixth decade [2,3].

The decrease in muscle strength and muscle mass contributes to the loss of the ability to live independently and thus becomes an important public health problem. Sarcopenia is associated with physical disability, poor physical performance, functional decline, falls, and hospitalization [4]. Multimorbidity is frequent in older individuals and some diseases, such as heart failure or chronic obstructive pulmonary disease, accelerate the loss of muscle strength and mass, creating a vicious cycle [5]. All these have a major impact on the patient′s quality of life [6]. Sarcopenia also increases the risk of falls. There is a high risk for hip fractures, as loss of muscle mass is frequently associated with loss of bone [5]. The high risk of falls in sarcopenic patients was found to be regardless of age, gender and other confounding factors [7]. In turn, falls are associated with functional deterioration, physical disability, impairment in activities of daily living, increased morbidity and mortality [8]. In a meta-analysis that included 17 studies, a significant association between sarcopenia and fractures was found, independent of study design, study population, gender, sarcopenia definition, geographical area or study quality [9].

Considering the specificities of older individuals, a sarcopenia-specific quality of life questionnaire (SarQoL) has been developed [10,11] and validated [12]. The Romanian version of the SarQoL^®^ was validated in 2017 [13]. Previous studies have reported the associated factors and the effects on the quality of life in adults with sarcopenia, using different diagnostic criteria. Only a few studies used the revised criteria of the European Working Group on Sarcopenia in Older People (EWGSOP2) [14,15,16]. The purpose of the present study was to evaluate the quality of life and the associated factors for sarcopenia in Romanian osteoporotic postmenopausal women, using the EWGSOP2 diagnostic criteria.

## 2. Materials and Methods

### 2.1. Study Design and Participants

Participants for this observational study were recruited from the postmenopausal women admitted to Medical Rehabilitation Clinical Hospital Băile Felix, România. To be selected, participants had to be previously diagnosed with primary osteoporosis (T-score ≤ −2.5, evaluated by DXA) and to have low hand grip strength. Low hand grip strength was defined according to the EWGSOP2 recommended cut-off of < 16 kg for women and was used to quantify the loss of muscle strength [1]. Criteria for exclusion were: (1) severe mobility disorders of the weight-bearing joints and cases with neurological conditions that affect balance and gait; (2) inability to walk for at least 10 min without a walking aid; (3) history of hip or knee arthroplasty; (4) inflammatory musculoskeletal conditions; (5) malignancies; (6) infectious diseases, (7) diabetic neuropathy; (8) cognitive impairments.

All participants provided written informed consent. The study complied with the Declaration of Helsinki and was approved by the Local Ethics Commission for Scientific Research of Medical Rehabilitation Clinical Hospital Băile Felix, România (4016/30.04.2018).

### 2.2. Assessments

Socio-demographic and clinical data (age, weight, height, body mass index, marital status, occupational status, years of menopause, history of and tendency towards falls, history of osteoporotic fractures, clinical conditions) were collected by interview and from medical documents. From the medical documents, the appendicular lean muscle mass determined by dual-energy X-ray absorptiometry was recorded for each participant in the study. Based on these results, and according to the recommended EWGSOP2 cut-off points for skeletal muscle mass index (appendicular lean mass/height^2^), the participants were categorized as having low muscle mass (<5.5 kg/m^2^) and normal muscle mass [1].

#### 2.2.1. Physical Performance

Physical performance was examined by the Timed Up&Go test, with the G-Walk system (BTS Bioengineering, Milan, Italy). It uses a validated wireless inertial sensor, made up of four inertial platforms, each composed of a tri-axial accelerometer, a tri-axial gyroscope and a magnetometer [17]. The G-sensor was attached to the participants fifth lumbar vertebra. The subjects were asked to stand up from a chair, to walk along a 3 m pathway at a self-selected speed, turn around and walk back to the chair and sit down. The recorded data were transmitted to the PC through a Bluetooth connection and processed by the BTS G-studio software (BTS Bioengineering, Milan, Italy). Women who scored ≥ 20 s were considered to have low physical performance.

Cases with low muscle strength were classified as having probable sarcopenia. We considered all participants that met the two EWGSOP2 diagnostic criteria-low muscle strength and low muscle mass—to have confirmed sarcopenia. Women with confirmed sarcopenia and low physical performance were categorized as having severe sarcopenia, according to the EWGSOP2 revised criteria [1]. We divided the study sample in two groups: group 1 comprised participants with probable sarcopenia (*n* = 58) and group 2, those with an established sarcopenia diagnosis, which included participants with confirmed and severe sarcopenia, according to EWGSOP2 (*n* = 64).

#### 2.2.2. Quality of Life

The quality of life was assessed using the validated Romanian version of SarQol questionnaire (Sarcopenia Quality of Life). This is a multidimensional questionnaire, evaluating seven domains of health-related quality of life—physical and mental health (D1), locomotion (D2), body composition (D3), functionality (D4), activities of daily living (D5), leisure activities (D6) and fears (D7) [10]. The 22 questions are rated on a 4-point Likert scale. Each domain is scored from 0 to 100 and an overall score is calculated. A higher score reflects a higher quality of life [12]. The SarQol questionnaire has good internal consistency and construct validity, good discriminative power and good responsiveness [12,13,14,18].

### 2.3. Statistical Analysis

The statistical analysis was performed using the Medcalc Statistical Software version 19.1 (MedCalc Software bv, Ostend, Belgium). All data were tested for normality with the Shapiro–Wilk`s test. Descriptive statistics were calculated for all socio-demographics’ characteristics (frequencies, means and standard deviation), SarQoL scores and TUG (median and interquartile range (IQR)). Between-groups differences were assessed using the independent *t*-test and Mann–Whitney test, respectively. Categorical data were compared using Chi-squared test. Logistic regression analysis was used to identify the factors associated with sarcopenia. Odds ratios (OR), 95% confidence intervals (CI) and *p* values were reported. Spearman rank correlation coefficient was used to assess the relationship between socio-demographic and clinical factors and the SarQoL scores. Variables that demonstrated significance were then entered into a stepwise multiple linear regression analysis to assess the predictors of quality of life, with SarQoL domains and total scores as a dependent variable. The significance level was set at *p* < 0.05 for all tests.

## 3. Results

The study sample comprised 122 women (mean age 67.02 ± 8.3 years) (ranging between 48 and 83 years) that met the inclusion criteria and agreed to participate in the study. More than half of the participants (52.46%) were diagnosed with confirmed and severe sarcopenia.

Table 1 summarizes the characteristics of the participants. There were no significant differences between the two groups in participants’ characteristics, except for weight, BMI and fall history. There was a higher percent of overweight and obese women in group 1 compared to group 2 (*p* < 0.0001). The proportion of overweight or obese women in our sample was 69.67%. A total of 93.10% of participants with probable sarcopenia and 48.43% of those with sarcopenia were overweight or obese. Women with sarcopenia had a higher frequency of history of falls than those with probable sarcopenia (*p* = 0.03).

The associations between the socio-demographic and clinical factors and the presence of sarcopenia were analysed by logistic regression. The factors significantly associated with sarcopenia were BMI (OR 0.79, 95%CI 0.71–0.88, *p* < 0.0001) and the history of falls (OR 2.84, 95%CI 1.13–7.09, *p* = 0.003). After adjusting for covariates (age, marital status, number of comorbidities and years since menopause), multiple logistic regression showed that BMI (OR 0.77, 95%CI 0.69–0.87, *p* < 0.0001) and the history of falls (OR 3.95, 95%CI 1.38–11.29, *p* = 0.01) together can predict the sarcopenic status. A lower BMI associated with at least one fall in the past would predispose osteoporotic postmenopausal women to sarcopenia.

Table 2 presents the total scores, as well as each domain scores of the SarQoL questionnaire. The D1, D4, D5, D7 and total SarQoL scores were significantly lower in women from group 2 compared to group 1.

In the whole study sample, significant lower scores were observed for D1, D4, D5, D7 and total SarQoL scores in the > 70 years group compared to the other two age groups, and for D2 and D3 in the > 70 years compared to the < 60 years group. In the probable sarcopenia group, no significant differences in all the SarQoL scores were observed between age groups. In group 2, women older than 70 years had significantly lower values for D1, D3, D4, D6 and total SarQoL scores than those from the other two age groups. For the D5 and D7 domains, women from group 2, older than 70 years, had significantly lower scores than those younger than 60 years (*p* < 0.05). When comparing the SarQoL scores between the two groups based on age, significantly lower scores were recorded only in the >70 years old group for D3, D4, D5 and total scores.

Physical performance did not differ significantly between the two groups. Low physical performance assessed with TUG (TUG > 20 s) was observed in 28 women from group 1 (48.27%) and in 34 women from group 2 (53.12%). According to the EWGSOP2 criteria, 53.12% women from group 2 were classified as having severe sarcopenia when using TUG performance. No age differences were observed between those with confirmed sarcopenia and those with severe sarcopenia.

In the whole study sample and in the probable sarcopenia group, significant greater TUG scores were observed in women older than 70 years compared to those younger than 60 years (21.8(17.9–30.6) vs. 16.3(13.3–21.38) s, *p* = 0.001 for the whole sample; 25.10(19.06–33.3) vs. 13.90(9.54–17.18) s, *p* < 0.001 for the probable sarcopenia group).

Significant negative correlations were found between SarQoL domains and total scores and some of the socio-anthropometric data for the whole study sample (Table 3). The history of falls and the number of comorbidities were negatively correlated with all SarQoL scores, except the D6 domains.

The stepwise multiple linear regression analysis with SarQoL total score as a dependent variable revealed a negative association with age, history of falls and being sarcopenic (adjusted *R*^2^ = 0.238; F_3,118_ = 13.59, *p* < 0.0001). Being older, sarcopenic with at least one fall in the past would negatively affect the quality of life of osteoporotic postmenopausal women. Table 4 shows the results of the regression analysis for all the SarQoL scores. In all regression models, history of falls was negatively correlated with all quality of life questionnaire domains, indicating that osteoporotic postmenopausal women with low muscle strength and falls in the past will have a poorer quality of life.

## 4. Discussion

The main aim of this study was to assess the relationship between sarcopenia and the quality of life in osteoporotic postmenopausal women. Both sarcopenia and osteoporosis are major health problems in postmenopausal women, negatively affecting the quality of life [19,20,21], the incidence of falls, and mortality [22,23,24,25]. To the best of our knowledge, there are no studies investigating the quality of life in Romanian postmenopausal osteoporotic women diagnosed with sarcopenia according to the updated EWGSOP diagnostic criteria.

There are several definitions, diagnostic criteria and cut-offs used for the diagnosis of sarcopenia [1,26,27,28,29,30,31]. In our study, we used the revised EWGSOP2 criteria. The percentage of confirmed and severe sarcopenia in osteoporotic postmenopausal women aged between 48 and 83 years at the time of assessment was 52.46%. Similar results were also found in other studies, showing the association of sarcopenia and osteoporosis [32,33,34,35,36,37]. Walsh et al., reported a similar prevalence of sarcopenia of 50% in osteoporotic postmenopausal women, using the loss of muscle mass for the sarcopenia diagnosis [38]. Hamad et al. found that sarcopenia was present in 74.6% of postmenopausal women with osteoporosis, supporting the results of Yoshimura that osteoporosis increases the risk of sarcopenia [39,40]. Studies indicate that the prevalence of sarcopenia increases with age [41,42]. In our study, the percentage of osteoporotic postmenopausal women diagnosed with sarcopenia increased with age, with 43.75% of women being older than 70 years. The true prevalence of sarcopenia cannot be correctly estimated, since various definitions, cut-offs or populations were used across studies.

History of falls and BMI were significantly associated with the presence of sarcopenia in osteoporotic postmenopausal women. Our results showed that osteoporotic postmenopausal women with at least one fall in the past had a significantly higher risk of developing sarcopenia. Similar results were presented by Clynes et al., who reported an association of falls in the last year and sarcopenia, diagnosed using the IWGS (International Working Group of Sarcopenia) definition, but not the EWGSOP one [43]. In their meta-analysis, Yeung et al. also reported a positive association between sarcopenia and falls [9]. Sepulveda-Loyola et al. found a strong association between osteosarcopenia (defined as the concomitant presence of osteoporosis/osteopenia with sarcopenia [44]) and falls and fractures history in community-dwelling older adults [45]. Other prospective studies have reported the association between sarcopenia and the incidence and risk of falls [7,46,47,48].

We found that BMI was lower in sarcopenic women than in those with probable sarcopenia from group 1. There was a higher percent of overweight and obese women with probable sarcopenia compared to those with an established sarcopenia diagnosis. In the sarcopenic group, we identified 14.06% cases of sarcopenic obesity. In recent years, the prevalence of obesity combined with sarcopenia had increased, resulting in a high-risk geriatric syndrome. Affected individuals are at risk of synergistic complications from both sarcopenia and obesity [49].

The logistic regression results in our study showed that osteoporotic postmenopausal women with a higher body mass index had a significantly reduced risk of developing sarcopenia. Similar results were found in previous studies [50,51,52], although in these studies the comparisons were made with non-sarcopenic subjects. Other studies also reported the protective effect of high body mass against sarcopenia in Asian population [53,54,55]. Moreno-Aguilar et al. found that a higher BMI represents a protective factor against the presence of osteosarcopenia [56]. Despite these findings, in a recent meta-analysis Shen et al. suggested, as well as Gonzales et al. in 2017, that BMI should not be used for making clinically important decisions at the individual patient level, since it could not differentiate between body weight components (body fat and lean mass) [57,58].

The SarQoL questionnaire is a specific health-related quality of life questionnaire for sarcopenia and muscle impairments [59]. Previous studies have demonstrated the ability of SarQoL to discriminate sarcopenic individuals with regard to their quality of life, as long as for the diagnosis of sarcopenia both muscle mass and muscle strength criteria were used [12,13,59,60,61]. The present study showed that osteoporotic postmenopausal women with probable and established sarcopenia had a reduced quality of life, as assessed with the SarQoL questionnaire. Our results were slightly lower than those obtained in previous studies by the sarcopenic participants [12,14,59,60,61,62]. We have to mention that, in previous studies, the EWGSOP criteria were used for establishing the diagnosis of sarcopenia, with a few exceptions where the revised EWGSOP2 criteria were used [14,62,63]. We found that the domains of physical and mental health (D1), functionality (D4), activities of daily living (D5), fears (D7) and total SarQoL scores were significantly lower in women with sarcopenia than those with probable sarcopenia. For locomotion (D2), body composition (D3) and leisure activities (D6) domains we have not found significant differences between sarcopenic groups. Similar results were found for the D6 domain in the study of Gasparik et al., with no significant differences between sarcopenic and non-sarcopenic participants when using the Romanian version of the SarQoL, as well as in the study of Konstantynowicz et al., who used the Polish version of the SarQoL [13,60]. The reason could be due to the fact that Romanian and Polish older people are not involved in many leisure activities [60].

In our study, osteoporotic postmenopausal women with sarcopenia who were older than 70 years had significantly lower values for physical and mental health (D1), body composition (D3), functionality (D4), leisure (D6) and total SarQoL scores than the younger ones. In the probable sarcopenia cases, the SarQoL scores were not influenced by age.

The negative impact of sarcopenia on quality of life has been largely investigated, although different criteria and questionnaires were used. The physical function domain of the quality of life has been proved to be impaired in sarcopenic patients, as assessed by the SF-36 questionnaire [52,64,65,66,67].

The multiple regression analysis in the present study showed a significant impact of age, history of falls and the presence of sarcopenia on the overall quality of life of postmenopausal osteoporotic women, as assessed with the SarQoL questionnaire. Older osteoporotic postmenopausal women with previous falls were more likely to have lower scores on physical and mental health (D1), functionality (D4) and activities of daily living (D5) domains. In association with the history of falls, the number of comorbidities was found to be a predictor only in the physical and mental health domain (D1) and body composition domain scores, respectively. Years since menopause, along with the history of falls, negatively influenced the fear domain (D7) score.

Several limitations of this study should be addressed. The study sample comprised only osteoporotic postmenopausal women with low grip strength, and no control group (premenopausal, non-sarcopenic) was included. Another issue that has to be mentioned is that the number of comorbidities was quite high and could influence the quality of life. The sample could have also been biased compared to the normal population, since the subjects were recruited from a rehabilitation clinic.

## 5. Conclusions

In summary, in our sample of osteoporotic postmenopausal women, the frequency of sarcopenia, as defined with the EWGSOP2 criteria, was relatively high. The body mass index and the history of falls could predict, together, sarcopenia in osteoporotic postmenopausal women. Our results showed that osteoporotic postmenopausal women with at least one fall in the past and a lower body mass index had a significantly higher risk of developing sarcopenia. History of falls and the number of comorbidities were negatively correlated with all quality of life questionnaire domains, indicating that postmenopausal women with low muscle strength and falls in the past will have a poorer quality of life. Older osteoporotic postmenopausal women, with previous falls and a confirmed sarcopenia diagnosis (low muscle strength and low muscle mass) were more likely to have a decreased quality of life. Future studies are required to identify women at risk, in order to reduce the prevalence of sarcopenia and its negative effects.

## Figures and Tables

**Table 1:** Socio-Demographic and Clinical Characteristics.

	All (*n* = 122)	Group 1 (*n* = 58)	Group 2 (*n* = 64)	*p*
Age, years	67.02 ± 8.03	66.48 ± 7.76	67.5 ± 8.79	NS
<60 years	26 (21.31)	10 (17.24)	16 (25)	NS
60–69 years	49 (40.16)	29 (50)	20 (31.25)	
>70 years	47 (38.53)	19 (32.76)	28 (43.75)	
Weight, kg	67.82 ± 11.02	72.34 ± 9.56	63.72 ± 10.69	<0.0001
Height, cm	157.93 ± 6.19	158 ± 6.18	157.9 ± 6.25	NS
BMI, kg/m^2^	27.22 ± 4.28	29.07 ± 3.71	25.55 ± 4.1	<0.0001
Underweigth (<18.5 kg/m^2^)	3 (2.46)	1 (1.72)	2 (3.13)	<0.0001
Normal (18.5–24.9 kg/m^2^)	34 (27.87)	3 (5.17)	31 (48.44)	
Overweight (25–29.9 kg/m^2^)	55 (45.08)	33 (56.9)	22 (34.37)	
Obese (>30 kg/m^2^)	30 (24.59)	21 (36.21)	9 (14.06)	
Years of menopause	19.66 ± 9.1	19.21 ± 8.25	20.08 ± 9.85	NS
Tendency to fall	55 (45.08)	24 (41.38)	31 (48.44)	NS
Fall history	28 (22.95)	8 (13.79)	20 (31.25)	0.02
Osteoporotic fractures history	30 (24.59)	18 (31.03)	12 (18.75)	NS
Number of comorbitites	5.85 ± 2.07	6 ± 1.97	5.72 ± 2.17	NS
Education				NS
Primary education (<8 classes)	52 (42.63)	23 (39.66)	29 (45.31)	
High school	50 (40.98)	27 (46.55)	23 (35.94)	
University	20 (16.39)	8 (13.79)	12 (18.75)	
Marital status				NS
Married	73 (59.84)	39 (67.24)	34 (53.13)	
Single	49 (40.16)	19 (32.76)	30 (46.88)	
Occupational status				NS
Working	46 (37.7)	23 (39.66)	23 (35.94)	
Retired	76 (62.3)	35 (60.34)	41 (64.06)	
Physical performance				
TUG (s)	19.6 (15.17–25.5)	19.45 (14.75–25.13)	19.65 (15.65–26.74)	NS
TUG>20s	61 (50)	28 (48.27)	33 (51.56)	NS
Data are presented as mean ± SD, number (percentage) or median [IQR]				

**Table 2:** Results of the SarQol Questionnaire in the Three Age Groups.

SarQoL Domains	All (*n* = 122)	Group 1 (*n* = 58)	Group 2 (*n* = 64)	*p* ^a^
D1	52.20 (45.50–65.50)	56.65 (48.90–72.20)	54.10 (49.45–62.20)	0.01
<60 years	58.30 (51.38–75.50)	66.65 (54.68–79.13)	53.85 (48.9–69.73)	NS
60–69	57.80 (48.90–67.20)	58.90 (52.20–71.1)	55.50 (42.25–65.60)	NS
>70 years	47.80 (37.80–55.50) ^b,c^	52.20 (45.50–58.9)	47.80 (35.25–51.93) ^b,c^	NS
D2	55.60 (47.20–66.70)	56.95 (50–70.10)	55.60 (42.38–63.90)	NS
<60 years	59.70 (55.60–70.10)	68.05 (54.85–73.60)	58.30 (55.60–66)	NS
60–69	55.60 (50–68.05)	55.60 (50–72.20)	53.50 (47.20–63.90)	NS
>70 years	50 (38.90–61.10) ^c^	55.60 (50–63.90)	47.20 (31.28–61.10)	NS
D3	54.20 (45.80–66.70)	58.3 (48.95–66.07)	54.20 (41.70–65.65)	NS
<60 years	60.40 (53.15–70.80)	64.6 (50–68.78)	58.30 (54.20–70.80)	NS
60–69	58.30 (45.8–70.80)	58.30 (45.80–68.75)	58.30 (46.85–73.95)	NS
>70 years	50 (37.50–62.50) ^c^	50 (50–66.7)	45.80 (37.50–57.28) ^b,c^	<0.05
D4	63.50 (53.32–75)	67.3 (57.7–78.6)	59.60 (50–70.80)	0.01
<60 years	69.60 (66.35–78.65)	73.10 (67.3–80.38)	68.75 (62.75–77.85)	NS
60–69	63.50 (56.45–78.7)	63.50 (57.70–78.80)	63 (52.78–75.98)	NS
>70 years	55.80 (48.10–69.20) ^b,c^	65.40 (55.80–71.20)	50 (45.18–55.80) ^b,c^	<0.05
D5	48.25 (37.30–60.17)	53.30 (43.30–66.25)	43.30 (33.30–55.60)	0.001
<60 years	56.70 (44.58–66.65)	59.15 (56.28–80.53)	51.65 (43–63.65)	NS
60–69	48.30 (41.70–63.80)	51.70 (42.50–66.30)	48.30 (34.20–61.45)	NS
>70 years	40 (33.30–50) ^b,c^	46.70 (38.30–60.70)	35.85 (30.83–45.45) ^c^	<0.05
D6	33.30 (16.60–33.3)	33.30 (16.60–52.85)	33.3 (16.6–33.3)	NS
<60 years	33.30 (16.60–37.45)	33.30 (12.45–41.60)	33.30 (33.30–45.75)	NS
60–69	33.30 (33.30–55.80)	33.30 (16.60–66.50)	33.30 (33.30–33.30)	NS
>70 years	33.30 (0–33.30) ^b^	33.30 (16.60–33.30)	24.95 (0–33.30) ^b,c^	NS
D7	87.50 (75–87.50)	87.5 (84.38–100)	87.50 (75–87.50)	0.006
<60 years	87.50 (87.50–100)	87.5 (87.50–100)	87.50 (77.08–96.88)	NS
60–69	87.5 (75–100)	87.5 (81.25–100)	87.50 (75–87.50)	NS
>70 years	75 (62.50–87.50) ^b,c^	87.5 (75–87.50)	75 (62.50–87.50) ^c^	NS
Total	55.45 (46.57–65.10)	57.90 (51.23–67.23)	53.33 (44.23–59.20)	0.003
<60 years	60.75 (54.30–68.05)	65.30 (59.33–76.2)	59 (54.30–66.98)	NS
60–69	56.30 (49.85–66.45)	59.90 (51.45–67.85)	56.30 (46.15–64.60)	NS
>70 years	48.10 (39.60–57.80) ^b,c^	56.10 (48.10–59.90)	45.25 (38.75–52.75) ^b,c^	<0.05

Data are presented as median and (IQR); *p* ^a^ relates to group 1–group 2 comparison (*p* < 0.05); ^b^ relates to the > 70 years and 60–69 years comparison (*p* < 0.05); ^c^ relates to the >70 years and <60 years (*p* < 0.05).

**Table 3:** Correlations between Sarqol Domaines and Clinical Variables.

	SarQoL D1	SarQoL D2	SarQoL D3	SarQoL D4	SarQoL D5	SarQoL D6	SarQoL D7	SarQoL Total
Age	−0.339 *	−0.238	−0.141	−0.318 *	−0.339 *	−0.062	−0.392 *	−0.392 *
BMI	−0.374 *	−0.171	−0.196	−0.302 *	−0.207	−0.184	−0.214	−0.297 *
Years of menopause	−0.379 *	−0.205	−0.153	−0.264 *	−0.303 *	0.017	−0.323 *	−0.314 *
No of comorbidities	−0.455 *	−0.312 *	−0.425 *	−0.396 *	−0.305 *	−0.204	−0.307 *	−0.381 *
Tendency to fall	−0.110	−0.113	−0.092	−0.077	−0.006	−0.059	−0.197	−0.086
Falls history	−0.406 *	−0.315 *	−0.344 *	−0.330 *	−0.263 *	−0.148	−0.361 *	−0.372 *
Osteoporotic fractures history	−0.061	0.08	−0.008	−0.059	−0.057	−0.150	0.005	−0.03
TUG	−0.206	−0.19	−0.137	−0.217	−0.236	0.053	−0.307 *	−0.244

Data represents the Spearman correlation coefficient; * *p* < 0.05

**Table 4:** Multiple Linear Regression Analysis with the Total and the Seven Domain Scores of SarQoL as Dependent Variables.

Independent Variable	B	SE	Beta	T	*p*	R^2^	Adjusted R^2^	Model Significance
SarQoL Total Score								
1. Age	−0.443	0.149	−0.268	−3.023	0.003	0.256	0.238	F_3,118_ = 13.59 *p* < 0.0001
2. Fall history	−10.318	2.946	−0.306	−3.502	0.0001			
3. Sarcopenia (confirmed and severe)	−5.140	2.365	−0.196	0.031	0.03			
SarQoL D1								
1. Age	−0.425	0.17	−0.222	−2.483	0.01	0.248	0.228	F_3.118_ = 12.97 *p* < 0.0001
2. Number of comorbidities	−1.454	0.66	−0.198	−2.202	0.02			
3. Fall history	−11.562	3.262	−0.310	−3.544	0.0006			
SarQoL D2								
1. Fall history	−15.035	3.608	−0.355	−4.167	0.0001	0.126	0.119	F_1,120_ = 17.3 *p* = 0.0001
SarQoL D3								
1. Number of comorbidities	−1.628	0.653	−0.222	−2.491	0.01	0.135	0.116	F_2,119_ = 9 *p* = 0.0002
2. Fall history	−9.679	3.208	−2.66	−3.017	0.003			
SarQoL D4								
1. Age	−0.474	0.145	−0.286	−3.259	0.001	0.261	0.249	F_2,119_ = 21.07 *p* < 0.0001
2. Fall history	−12.440	2.865	−0.369	−4.341	<0.0001			
SarQoL D5								
1. Age	−0.540	0.190	−0.252	−2.838	0.006	0.189	0.168	F_3,118_ = 9.186 *p* < 0.0001
2. Fall history	−7.976	3.820	−0.188	−2.088	0.01			
3. Sarcopenia (confirmed and severe)	−7.522	3.066	−0.220	−2.453	0.01			
SarQoL D6								
1. Fall history	−12.402	4.671	−0.235	−2.655	0.009	0.055	0.047	F_1,120_ = 7.04 *p* = 0.009
SarQoL D7								
1. Years of menopause	−0.458	0.137	−0.293	−3.344	0.001	0.187	0.174	F_2,119_ = 13.75 *p* < 0.0001
2. Fall history	−8.802	2.954	−0.263	−2.980	0.003			
